# The Practical Challenges of Evaluating a Blanket Emergency Feeding Programme in Northern Kenya

**DOI:** 10.1371/journal.pone.0026854

**Published:** 2011-10-26

**Authors:** Andrew Hall, Moragwa Oirere, Susan Thurstans, Assumpta Ndumi, Victoria Sibson

**Affiliations:** 1 Centre for Public Health Nutrition, School of Life Sciences, University of Westminster, London, United Kingdom; 2 Save the Children UK, Nairobi, Kenya; 3 Save the Children UK, London, United Kingdom; Aga Khan University, Pakistan

## Abstract

A blanket supplementary feeding programme for young children was implemented for four months in five northern districts of Kenya from January 2010 because of fears of food insecurity exacerbated by drought. An attempt to evaluate the impact of the food on children's anthropometric status was put in place in three districts. The main aim of the analysis was to assess the quality of the data on the cohort of children studied in the evaluation and to propose methods by which it could be improved to evaluate future blanket feeding programmes. Data on the name, age, sex, weight and height of a systematic sample of children recruited at 61 food distribution sites were collected at the first, second and third rounds and again at an extra, fifth food distribution, offered only to the evaluation subjects. Of the 3,544 children enrolled, 483 (13.63%) did not collect a fifth ration. Of the 2,640 children who were considered by their name to be the same at the first and fifth food distribution (13% were different), data on only 902 children (34.17%) were considered acceptable based on their age (an arbitrary ±3 months different) and their length or height (between >−1 or ≤4 cm different) at the two instances they were seen. Data on nearly two thirds of children were of questionable quality. The main reasons for the poor quality data were inconsistencies in estimating age or because caretakers may have brought different children. Recommendations are made about how to improve data quality including ensuring that entry to a blanket feeding programme is clearly based on height, not age, to avoid misreporting age; careful identification of subjects at all contacts; and using well-trained, specialist evaluation staff.

## Introduction

Many humanitarian agencies provide supplementary food for young children during an emergency to try to prevent weight loss and malnutrition. In order to estimate the effect of supplementary food on weight change it is necessary to have an unfed control group to assess what would have happened without the food and calculate the probability that any difference in mean values is statistically significant. For example, if the supplementary food simply sustained children's body weight whereas without it they would have lost weight, the effect of the food is undetectable without having an unfed control group. Simply measuring children before and after supplementary feeding cannot estimate the effect on body weight that is attributable to the supplementary food, particularly if the mean body weight does not change. As an unfed control group is obviously unethical during a humanitarian emergency, the only option is to collect data from natural observations to provide indirect but plausible evidence of impact [1]. This poses substantial challenges for agencies trying to account for the effectiveness and cost-effectiveness of any form of humanitarian aid, including supplementary food.

**Figure 1:**
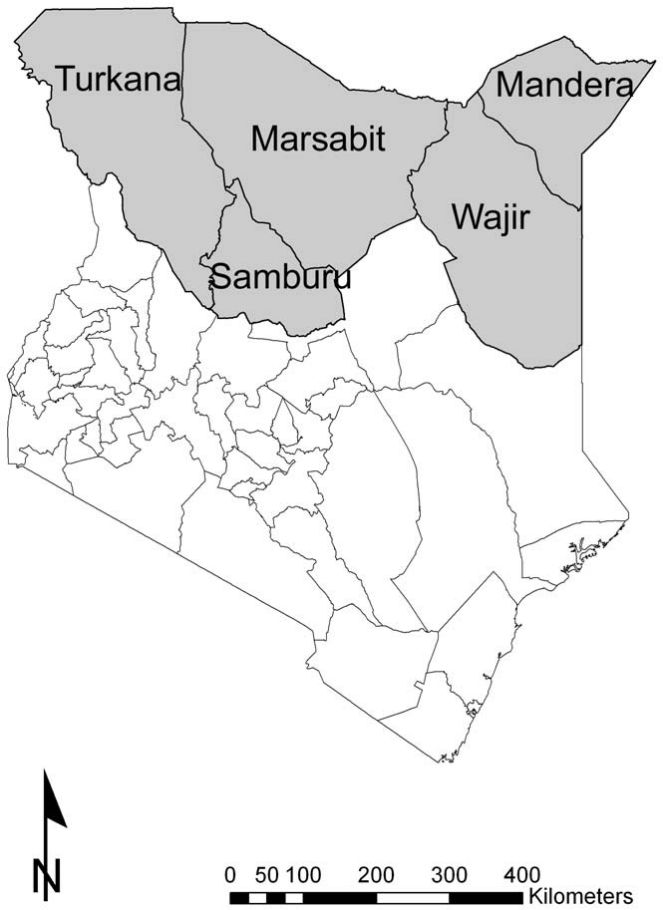
A map showing the five districts of Kenya in which the blanket supplementary feeding programme was implemented in January 2010.

A mass or ‘blanket’ supplementary feeding programme was implemented in five northern districts of Kenya between January and April 2010 because of fears for an increase in the incidence of malnutrition as a result of seasonal food insecurity exacerbated by persistent drought. The five programme districts of Mandera, Marsabit, Samburu, Turkana and Wajir cover 45% of Kenya's total land area (Figure 1) but at the time contained only 4.5% of the population of 28.87 million recorded in the 1999 census [2]. The primary stated aim of the programme was to protect the nutritional status of an estimated 300,000 children aged 6–59 months, or 20% of the 1999 census population [3]. All children <110 cm in height were eligible for a ration of food plus any taller children whose mother insisted that they were <60 months of age [3], [4]. This height is the median value for boys aged 60 months in the World Health Organization (WHO) reference tables [5].

Rations of food provided by the World Food Programme were given on four occasions, each about 30 days apart, beginning in January 2010. They consisted of 7.5 kg of corn-soy blended flour and 0.75 kg of vegetable oil to provide an average of 1,000 kcal/day/child. The food was distributed by non-governmental agencies (NGOs) at the sites of pre-existing feeding programmes and at some extra sites, to improve local access.

The aim of the evaluation was to try to detect evidence for an effect of the rations on the anthropometric status of children in three main ways. First, by comparing the anthropometric indices of newly recruited children at the second and third food distributions with children enrolled at the first distribution, to assess if their anthropometric status was getting worse during a period when food security was supposedly poor or deteriorating. Second, by comparing the weight change of children who received two, three or four rations of food during the programme, in order to detect a dose-response relationship. Third, by comparing the weight change of singleton children with children matched for age and sex in households of two or more children, based on the premise that if a ration was shared it would be less effective than if it was given to an only child, and assuming that the ration was not shared outside the household. Historical data were also collected to examine trends in mean z-scores and the prevalence of undernutrition in the same communities, but they are not discussed here.

The aim of the analysis presented here was to assess the quality of the data collected on the cohort of children studied in the evaluation, to identify reasons for its poor quality, and to propose methods by which it could be improved to evaluate future blanket feeding programmes.

## Methods

The rations of food were distributed initially at 540 sites in the districts of Mandera (99 sites), Marsabit (55), Samburu (101), Turkana (162) and Wajir (123) (Figure 1) by a group of eight NGOs led by Save the Children, UK (SCUK).

An arbitrary number of 25 food distribution sites were randomly selected for study in each of two adjacent districts, Mandera and Wajir, 26 sites operated by SCUK and 24 by Islamic Relief. At the request of the National Nutrition Technical Forum, 25 sites were also randomly selected in Turkana district, which is in a different livelihood zone and contains a different ethnic group, the Turkana; most people in Mandera and Wajir are Somali. The Forum wanted to know if there were differences in impact on children in the two locations. Four agencies were responsible for collecting data in Turkana: Merlin (10 sites), Samaritan's Purse (4), IRC (1) and World Vision (10). Because of a delay in funding, World Vision did not collect data at their 10 sites. The staff of each NGO was responsible both for distributing the food and for collecting data for the evaluation. All members of staff were given one day's theoretical training by SCUK on the blanket feeding programme, community mobilisation, organising distribution sites and on the evaluation methods, including sampling children and administering questionnaires. The five NGOs then organised two days practical training for their field staff according to an agenda specified in the programme guidelines [3]. All the NGO staff were nurses or nutritionists who were supposedly practiced at making anthropometric measurements, so no specific training on anthropometry was arranged.

The aim was to recruit up to a 10% sample of children at the first food distribution at each study site and then at the same sites recruit all new children who were brought to claim a ration at the second and third food distributions, as they should not yet have received any supplementary food. As there were no prior data on how many children might attend each site, the sampling interval was determined at the start of each food distribution based on the number of children who turned up or who were expected by local leaders. If it was <1000 children, then a sampling interval of 10 was used: a number from 1 to 10 was taken from a random numbers table, a child in the first ten was selected using that number, then every 10^th^ subject was recruited systematically. If 1,000–2,000 children were expected to claim a ration, then a sampling interval of 20 was applied in the same way.

A power calculation using Stata 11 [6] indicated that a sample size of 3,022 children could detect a 4% difference in the prevalence of wasting from 26% (the average prevalence reported in three previous surveys) [7] over the period of intervention allowing for a design effect of 2 due to the clustering of children around distribution sites, 25% drop-out, and assuming a power of 80% and a two-sided statistical significance of *P*<0.05.

The evaluation was approved by the Kenya Ministry of Public Health and Sanitation as a part of their humanitarian services and by the Ethical Review Committee of the University of Westminster in London.

A statement explaining the evaluation was read to the caregiver of each selected child who then signed the data form or provided a thumb print if unable to write, in acknowledgement. The caregiver was told that they could collect an extra, fifth ration, one month after the fourth and final ration for all beneficiaries. This acted as an incentive to bring back the child to be weighed after the fourth ration had been consumed, and so estimate the effect of all four standard rations.

Each child was weighed to a precision of 0.1 kg on electronic scales (Uniscale, UNICEF) and measured to a precision of 0.1 cm, supine if <87 cm and standing if ≥87 cm on locally made stadiometers, according to Kenya Government guidelines [3].

The mother was interviewed and asked her name, her child's name, her village name, and the number of other children she had, including any other children aged less than 5 y old. Any caregiver who was not the mother was asked about the child's family to obtain the same answers.

The sex of the child was noted and the date of birth was recorded from a document, such as a health card or birth certificate; or it was given by the mother or caregiver; or, if the caregiver did not know, it was estimated using a local calendar of events. Other data were recorded at the interview about the mother's household circumstances, on access to food from other programmes and on her child's recent health, but they are not reported here. The data sheets were sent to Nairobi for computer data entry after each round, and data were entered only once.

Each caregiver was given a ration card for the child with a unique identification number created from the site code and the child's serial number. These numbers were also recorded in a register book for each site and on the data forms for each child at each visit to collect a ration and were used to link data. Ration cards were given only at sites taking part in the evaluation.

When the mother or caregiver returned to collect the fifth, extra ration, the child was weighed and measured again to estimate any difference and the date of birth was also recorded again, using the same methods, to assess how it agreed with the date estimated at enrolment. A small survey of children who did not collect the fifth ration was done, but is not reported here.

The date of birth and date of visit were used to estimate each child's age in months at enrolment and z-scores of height-for-age, length-for-age and weight-for-height were calculated using a macro for Stata 11 [6] published by the WHO [8]. This flags values of weight-for-age that are >5 and <−6 S.D., values of height-for-age that are >6 and <−6 S.D. and values of weight-for-height that are >5 and <−5 S.D. because the underlying data are likely to be wrong.

In order to assess the quality of the data, five indicators were used. First, the name of the child recorded on both occasions on different data forms; the names were judged to be the same, different or possibly different. Second, the age distribution of children aged 6 – 59 months, which should be similar to the distribution reported in the last district census. Third, the number of z-score values that were flagged by the WHO anthropometry macro in Stata. Fourth, the difference in months between the age estimated at the first and last visits; for the purposes of analysis a difference of ±3 months was arbitrarily taken to be acceptable. Fifth, the difference in length or height of each child between the first and last measurements. An acceptable range was taken to be −1 cm to +4 cm. This is a combination of measurement error and rounding (which was evident in the data) of ±1.0 cm; changes in measuring children from supine to standing of 0.7 cm; plus a possible gain in height of up to 2.7 cm rounded up to 3.0 cm, which is the maximum possible gain for a nearly 5 year old boy who is 3 S.D. above the median in height according to WHO growth references. A change greater than 4 cm or less than −1 cm should not have been possible.

Not all percentages may add up to 100% because of rounding.

## Results

Table 1 shows the number of sites and children who were enrolled in each district at each round of food distribution. Children were not enrolled at five randomly selected sites during the first round because the sites were unsafe. The mother was the respondent to the questionnaire for 91.95% of subjects. Documentary evidence of the date of birth was provided for 20.77% of 3,544 subjects while dates were reported by 44.92% of caregivers or estimated from a local calendar of events by 33.86%. Data were missing for 0.45% (16) of subjects.

**Table 1:** The number of sites where new children were enrolled at each round of food distribution and the number of children enrolled.

			Round of food distribution					
District			1	2	3	4		Total
Mandera	Sites		25	19	11	0		
	Children		907	236	59	0		1,202
Wajir	Sites		21^a^	24	22	5		
	Children		496	760	324	19		1,599
Turkana	Sites		15^b^	12	4	0		
	Children		614	93	36	0		743
Total	Sites		61	55	37	5		
	Children		2,017	1,089	419	19		3,544

a Five sites were unsafe to visit; ^b^ 10 sites were not included because funds were not available in time.

Of the 3,544 children enrolled, 483 (13.63%) did not return to collect a fifth ration. Of the 3,061 children who did return, 196 (6.40%) had a different name and 200 (6.53%) had a possibly different name, indicating that perhaps up to 13% of mothers had brought a different child to collect the last ration. There were 3 names missing.

**Figure 2:**
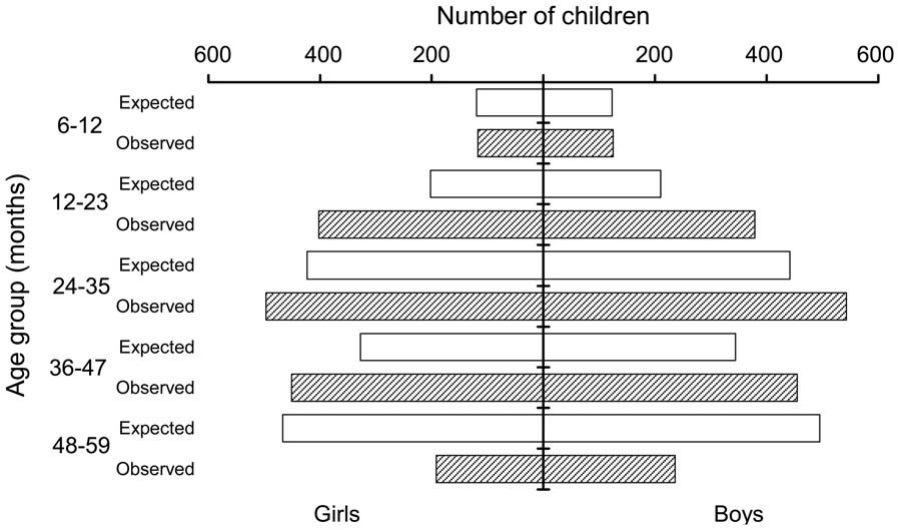
The observed and expected numbers of children (n  =  3,555) by age group and sex.

Figure 2 shows the age distribution of 3,397 children aged 6–59 months whose age was recorded at enrolment in comparison with the expected age distribution based on the 2009 census in the same three districts [9]. The expected number of children aged 6–11 months was estimated by dividing by two the numbers recorded for children aged 0–11 months. Figure 2 shows that there were 89% more children than expected aged 12–23 months and 56% fewer children aged 48–59 months, suggesting a bias towards younger children. Only 93 children (2.63%) were older than 60 months (not shown in Figure 2), which seems unlikely if the entry criterion to the programme was based on a height of <110 cm rather than age and should have included older but stunted children. There were no statistically significant differences in the mean reported age of children enrolled at the first, second or third food distributions.

The WHO macro to calculate anthropometric indices flagged baseline values of weight-for-height, height-for-age and weight-for-age for 237 children (6.67%) of which 67 (2.56%) were weight-for-height, suggesting that a measurement of weight or height was incorrect. The same values were flagged for fewer children at the fifth food distribution: 113 (3.18%) had any index flagged while 35 (1.17%) had the value of weight-for-height flagged.

**Figure 3:**
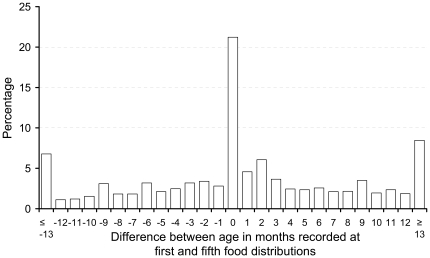
The distribution of the differences in ages of children in months estimated at the first and fifth food distributions.

Figure 3 shows the distribution of the difference in age in months recorded for 3,061 child at enrolment and at the fifth food distribution, an average of 97 days later (range 16–135 days), depending on when children were enrolled. Only 21.23% of children were recorded as having the same month of age; 23.72% were 1 to 3 months younger or older; 25.22% were 4 months or more younger; and 29.79% were four months or more older. In summary, 44.95% of children were within ±3 months of the same age and 55.05% were ≥4 or ≤4 months different in age.

**Figure 4:**
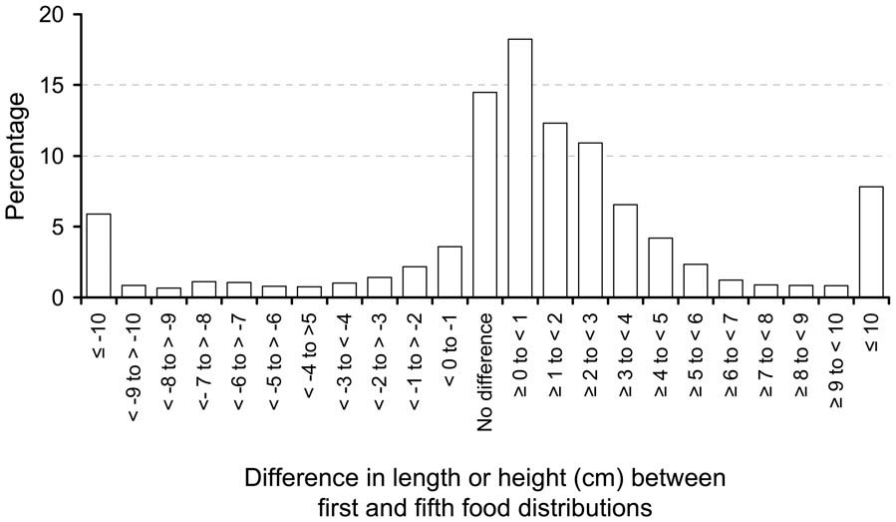
The distribution of the difference in height of children at the first and fifth food distributions.

Figure 4 shows the distribution of the difference in height of 3,032 children measured at enrolment and the fifth distribution of whom 66.09% were within a range of >−1 to <4 cm, 15.77% were >1 cm shorter, and 18.14% were ≥4 cm taller.

Of the 2,640 children who were considered by their name to be the same on both occasions, data on only 902 children (34.17%) were considered to be acceptable based on both their stated age (±3 months different) and length or height (>-1 or ≤4 cm different) at the two instances they were seen, so that data on nearly two thirds of children were of questionable quality. Because of these discrepancies, no further analysis was done to assess the impact of the feeding programme.

## Discussion

Any attempt to estimate the impact of supplementary food on weight gain requires that each child is measured twice, at the start and end of the programme to obtain paired measurements, and that accurate data on age is obtained if anthropometric indices other than weight-for-height are to be calculated. The data collected during the present evaluation in northern Kenya indicated that a large proportion of children were not the same at the first and fifth food distribution and that the age of many children was not given or estimated consistently so was probably inaccurate. There are a number of likely reasons for this: the possibility that mothers did not bring the enrolled child to collect the fifth and final ration; systematic bias or errors in estimating age; inconsistent estimates of age on separate occasions; and errors in making anthropometric measurements, in recording data, and in data entry.

The age distribution shown in Figure 2 is unlike a typical age pyramid and suggests that many mothers, who made up 92% of the caregivers at enrolment, were either not giving or not estimating correctly the age of their child, perhaps to ensure that they obtained a ration of food. The fact that about 40% of all children were either shorter by 1 cm or more or taller by 3 cm or more suggests that a substantial proportion of caregivers did not bring the same child to collect the fifth ration, although some differences could be measurement errors made by busy staff. The enrolment forms had been sent for data entry, so they could not be checked at the fifth food distribution, while the ration card only recorded each child's name and ID number. Although a few mothers gave the name of a different child at the fifth food distribution which could have been questioned, the fieldworkers assumed that the child who had been given a ration card was brought back to collect the final ration.

It is to be expected that parents will make every effort to obtain a ration of food for their children during a food shortage and the many years of food insecurity in this part of Kenya have led to a degree of dependency on humanitarian assistance. An eligibility threshold of <110 cm in height [4] was applied to try to eliminate a reason for parents to be untruthful about giving the correct age of their child. This did not seem to work, perhaps because community mobilisers did not understand or make it clear to parents, because mothers did not understand, or because mothers were mistrustful of a different criterion of eligibility for health services from the usual, which is age. This requires more research. But a recent survey by SCUK in Mandera and Wajir indicated that about 80% of children <2y old had a health card [10] compared with only 9.7% of mothers with children of the same age during the blanket feeding programme, suggesting that they were not keen to divulge the recorded date of birth.

Several things compromised data quality. First, the design and implementation was complicated by the request to evaluate the impact in a different a livelihood zone, among a different ethnic group, and by additional NGOs. This increased the sample size, increased the cost, and increased the number of agencies involved from two to six, with consequences for staff training and data quality. As this was the first attempt in Kenya to evaluate the impact of a blanket feeding programme using such methods, it might have been best to focus efforts in two contiguous districts among a single ethnic group and in one livelihood zone, to keep it as simple as possible.

Second, the pressure to begin distributing rations reduced the time available for training field staff of 6 NGOs to 2 days by 8 different trainers, adding other factors that may have compromised data quality.

Third, the personnel doing the evaluation were also responsible for registering and distributing rations to about 500–2,500 beneficiaries at each site during the first food distribution, so the staff were overburdened.

Fourth, supervising the collection of data at food distribution sites spread over an area of 150,000 km^2^ posed an insuperable problem to the lead NGO. Wajir town is 1,100 km by road to the main town in Turkana. So after being quickly trained, the NGO staff were unsupervised by the lead evaluation agency.

Whether the design could have provided evidence of impact is a separate issue that is hard to assess as the data were not good enough. A national programme of therapeutic and supplementary feeding during a humanitarian emergency in Burundi seemingly failed to reduce the prevalence of wasting, perhaps because only 55% coverage was achieved [11]. Three different lines of evidence suggested that supplementary feeding programmes during a war in Guinea-Bissau prevented in increase in the prevalence of undernutrition [12]. But even if the evidence of the impact of supplementary feeding programmes is inevitably indirect because having an unfed control group is impossible, the experiences described here offer useful lessons that could be applied to improve the quality of data in future evaluations of blanket feeding programmes in Kenya and elsewhere.

First, the evaluation should be put in place as the intervention is being planned so that the evaluation is a part of the programme, not an external component. Both require preparation, swiftness and adequate funding.

Second, community mobilisers need to explain clearly and effectively to potential beneficiaries the criterion for eligibility: height <110 cm, not age <59 months, the usual threshold for health programmes. Ideally this criterion would be used for all programmes because it is simple, objective and transparent, and would include stunted children older than 5y, who could also benefit from most interventions. The disadvantage of using height is that there is no easily calculated denominator to estimate both the numbers of eligible children and coverage, whereas a denominator based on age can be estimated from census data. As an estimate of coverage is important indicator of the effectiveness of an intervention, a separate survey would be necessary at additional expense [13], [14].

Third, every study child ideally should be identified either using a digital photograph or perhaps using a fingerprint reader, either in a personal digital assistant (PDA) or connected to a laptop computer, to confirm their identity at subsequent contacts. Battery powered PDAs could also be used to collect, store and compare data in the field, so that widely differing anthropometric measurements could be flagged and checked immediately. Such devices require a capital outlay, a software programmer and field testing before deployment, which is expensive, but could improve data quality and speed up the process of analysis and reporting as well as increasing the validity of the evaluation. If suitable equipment is not available, then key data should be copied onto ration cards to act as a check, including the estimated date of birth and the first height and weight. Neither process would guarantee that the same child is seen on all occasions, but any substitute could be identified on the spot.

Fourth, the staff doing the evaluation should be different from the staff delivering the ration cards, food or other interventions, so that both jobs are done as well as possible in an often chaotic and busy environment in which agitated parents demand attention. Ideally the evaluation staff should either be drawn from only one NGO or, preferably, should be separate from the agency delivering the food so as to remain objective and independent. A disadvantage of using an independent agency is that it does not build the capacity of NGOs to conduct robust evaluations. As the rations were delivered 30 days apart at each site and delivery was also staggered over 30 days, it would have been feasible to have employed a full-time evaluation team to collect data at up to 30 sites in each district. The number of 25 evaluation sites in each district was arbitrary, but when subjects are studied in clusters it is important statistically to have a sufficient number of clusters as well as sufficient subjects [15]. A dedicated evaluation team would require additional funding, an issue not addressed here, but the compromised evaluation also represents a waste of resources as well as the time of staff and mothers. The evaluation staff also require specialised training in anthropometric measurements, even if they have done them many times before, because both accuracy and precision are required, and should not be assumed. Training should be also given in interviewing mothers, ideally in the local language. A team consisting of 4 interviewers could interview about 100 mothers a day if an interview took 15 minutes, while two specialist anthropometry technicians could weigh and measure the children. As the food distribution sites in northern Kenya were far apart over rough terrain, every evaluation team also needs its own supervisor to attend to quality control.

Finally, data analysis should be done as quickly as possible, in the field, so that systematic errors such as rounding can be identified and rectified by re-training or reorganisation of procedures. If all data are entered in the field onto PDAs, the confirmation of each entry would duplicate the process of double data entry. Data could also be merged from different field teams and analysed quickly in the field using batch files written for statistical software.

By reporting the problems and lessons learned from this evaluation of a blanket feeding programme it is hoped that future evaluations will be better planned and implemented and may provide plausible evidence of a benefit to children's nutritional status of a blanket feeding programme.
